# Effects of Oxygen Prebreathing on Bubble Formation, Flow-Mediated Dilatation, and Psychomotor Performance during Trimix Dives

**DOI:** 10.3390/sports12010035

**Published:** 2024-01-22

**Authors:** Ivana Šegrt Ribičić, Maja Valić, Linda Lušić Kalcina, Joško Božić, Ante Obad, Duška Glavaš, Igor Glavičić, Zoran Valić

**Affiliations:** 1 Department of Pulmonary Diseases, University Hospital Center Split, 21000 Split, Croatia; ivana.segrt05@gmail.com; 2 Department of Neuroscience, University of Split School of Medicine, 21000 Split, Croatia; linda.lusic.kalcina@mefst.hr; 3 Department of Pathophysiology, University of Split School of Medicine, 21000 Split, Croatia; josko.bozic@mefst.hr; 4 Department of Health Studies, University of Split, 21000 Split, Croatia; ante.obad@ozs.unist.hr; 5 Department of Internal Medicine, University of Split School of Medicine, 21000 Split, Croatia; duska.glavas@gmail.com; 6 Department of Marine Studies, University of Split, 21000 Split, Croatia; igor.glavicic@gmail.com; 7 Department of Physiology, University of Split School of Medicine, 21000 Split, Croatia; zoran.valic@gmail.com

**Keywords:** scuba diving, oxygen, bubble formation, endothelial function, psychomotor performance, trimix

## Abstract

*Introduction:* This research was performed to examine the effects of air and oxygen prebreathing on bubble formation, flow-mediated dilatation, and psychomotor performance after scuba dives. *Methods:* Twelve scuba divers performed two dives using a gas mixture of oxygen, nitrogen, and helium (trimix). In a randomized protocol, they breathed air or oxygen 30 min before the trimix dives. Venous bubble formation, flow-mediated dilatation, and psychomotor performance were evaluated. The participants solved three psychomotor tests: determining the position of a light signal, coordination of complex psychomotor activity, and simple arithmetic operations. The total test solving time, minimum single-task solving time, and median solving time were analyzed. *Results:* The bubble grade was decreased in the oxygen prebreathing protocol in comparison to the air prebreathing protocol (1.5 vs. 2, *p* < 0.001). The total test solving times after the dives, in tests of complex psychomotor coordination and simple arithmetic operations, were shorter in the oxygen prebreathing protocol (25 (21–28) vs. 31 (26–35) and 87 (82–108) vs. 106 (90–122) s, *p* = 0.028). *Conclusions:* In the oxygen prebreathing protocol, the bubble grade was significantly reduced with no change in flow-mediated dilatation after the dives, indicating a beneficial role for endothelial function. The post-dive psychomotor speed was faster in the oxygen prebreathing protocol.

## 1. Introduction

Diving with a self-contained underwater breathing apparatus, known as scuba diving, has become a popular activity worldwide, although it is considered a high-risk form of sport or leisure activity [1]. Underwater scuba divers are faced with several possible risks when breathing pressurized gas mixtures. Inert gases, when breathed under elevated pressure, dissolve in increased amounts in body tissues and fluids. If divers surface at an inappropriate rate (e.g., too fast or without obligatory decompression stops), such gases do not remain in a dissolved state and form numerous tissue and vascular bubbles that may cause well-documented damage to different organ systems, known as decompression incidents, including arterial endothelial dysfunction [2] and changes in cardiovascular function [3,4,5]. Other effects, such as alterations in cognitive functions, motor control, and mood have also been reported in scuba diving [6,7,8].

Air and enriched air nitrox (EANx) are commonly used as breathing gases during recreational diving. Additionally, the gas mixture of oxygen (O_2_), nitrogen (N_2_), and helium (trimix) or switching gases during a decompression stop have been used to improve the safety and duration of technical dives. EANx consists of a lower N_2_ proportion and a higher O_2_ proportion in comparison to air, which is intended to decrease the risks of decompression sickness (DCS) and vascular bubble formation [9,10] and to prevent the narcotic effects of nitrogen [11,12]. Several studies have shown that breathing oxygen before a dive or during decompression could have protective effects and might be helpful in the prevention of DCS and in reducing bubble formation during recreational open-water air dives [13,14]. However, hyperoxia might have detrimental effects that lead to decreased flow-mediated dilatation (FMD), indicating the impairment of endothelial function [15]. On the other hand, a shorter duration of exposure to hyperoxia might lead to decreased bubble formation with the preservation of FMD [9]. Despite the increased popularity of trimix as a breathing mixture for technical dives, no study has reported the effects of O_2_ prebreathing on bubble formation and endothelial function during deep technical dives using trimix.

Another noteworthy aspect is that while certain studies have proposed positive effects of scuba diving [16], numerous research findings indicate cognitive impairment among scuba divers, posing a potential threat to diving safety [17,18]. Additionally, the short-term detrimental effects of diving have been shown through an increase in response time and a decrease in sustained attention shortly after scuba diving, indicating an impairment of cognitive functions in the period after a dive [8]. The measurement of cognitive function before, during, and 30 min after a dive employing behavioral computer-based testing showed an improvement in cognitive abilities upon arrival at 0.4 MPa, followed by a progressive decline that lasted up to 30 min after the dive [6]. Furthermore, it seems that a higher fraction of inspired oxygen had a positive effect on mental arousal and cognitive performance [6,7,19]. Although previous research clearly showed the short-term effects of a single dive on psychomotor performance, detailed information about the different diving protocols and gas mixtures used, as well as the potentially beneficial effects of oxygen, either in the breathing gas mixture or in prebreathing protocols, is still needed for a better understanding of the cognitive and psychomotor performance and possible protective procedures during technical open-water dives. The Complex Reactiometer Drenovac test battery (CRD), a psychodiagnostic chronometric instrument, enables the measurement of simple and complex cognitive and psychomotor performances, providing data on the speed of mental processing and the stability of psychomotor functioning during the process of testing. The CRD can be used repeatedly on the same participants and will yield the same test results. Additionally, the instrument is applicable to participants of all ages, without language restrictions or requirements for other specific knowledge. CRD tests have been used to estimate psychomotor performances in diverse study designs [20,21,22]. Psychomotor performance during diving can be affected by various factors such as cold water, ambient pressure, and anxiety [23,24], justifying the need for a well-controlled study with precise information on the water temperature, dive depth, and anxiety level. Additionally, an individual’s endothelial function should be taken into consideration due to its association with a reduced speed of information processing, psychomotor skills, mental flexibility, and attention [25,26].

The objective of this study was to investigate whether prebreathing 100% oxygen influences the formation of venous gas bubbles, endothelial function, and cognitive and psychomotor performance during 60 msw open-water dives using trimix. The first hypothesis was that bubble formation would be decreased in the protocol with the prebreathing of 100% oxygen compared to the prebreathing of air prior to trimix dives. More importantly, we wanted to test the hypothesis of whether endothelial function would be impaired by this procedure. Furthermore, the purpose of this study was to assess the short-term effects of scuba diving on cognitive and psychomotor performance, which were determined based on participants’ reaction times during computer-based psychomotor testing 30 min after a dive. We additionally assessed the level of anxiety of the scuba divers.

## 2. Materials and Methods

### 2.1. Subjects and Study Design

**Figure 1:**
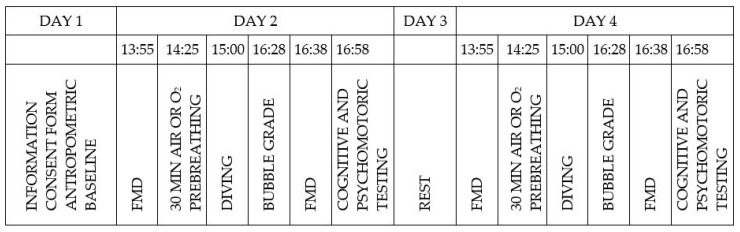
Experimental protocol showing activities of the divers in the study and indicating diving on day 2 and day 4. Prebreathing of air and oxygen was randomly assigned.

This study was performed with twelve scuba divers at the Big Blue Diving Center in Bol, on the island of Brač, Croatia. The subjects were certified (PSS trimix 60 M or similar) scuba divers with experience of more than 20 h of diving per year who were trained to dive trimix to 60 msw. The divers included in this study never suffered from decompression sickness (DCS). The exclusion criteria were divers without appropriate certification; divers under 18 years of age; and divers with adverse medical conditions, such as heart failure, coronary heart disease, malignant disease, or any history of neurological diseases. The divers were asked to refrain from tobacco and alcohol usage for at least 6 h before the dive. All dives were scheduled at 3 pm, with the bottom sea water at about 17 °C and outside temperatures ranging from 29 to 34 °C. Each diver performed two dives 48 h apart, and they served as their own controls for the analysis. The order of the diving protocols, using different pre-dive gases, was randomized. In protocol 1, the divers breathed air from the environment 30 min before a trimix dive (air prebreathing); in protocol 2, the divers breathed normobaric 100% oxygen from a compressed tank 30 min before a trimix dive (O_2_ prebreathing). The divers used a trimix breathing mixture (20% O_2_, 30% He, and 50% N_2_) for a descent to 60 msw and an ascent to 21 msw, at which point they changed to nitrox 50 (50% O_2_ and 50% N_2_), which was used for decompression until reaching the surface. Dives were performed as open-circuit dives with each diver using his/her main personal equipment and 7 L stage tanks provided by the diving center. The experimental protocol is depicted in Figure 1.

**Figure 2:**
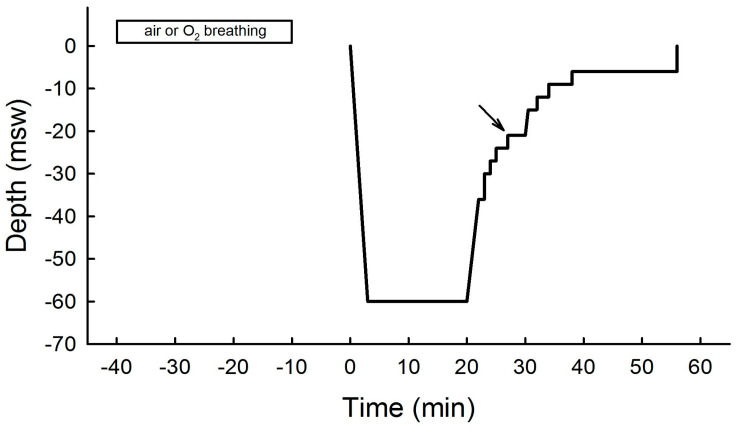
Diving protocol using trimix mixture (20% O_2_, 30% He, and 50% N_2_) for descent and ascent to 21 msw and using nitrox 50 (50% O_2_ and 50% N_2_) for decompression until resurfacing according to the protocol given by V−planner. Rectangle represents 30 min of air or oxygen prebreathing at the surface. Arrow denotes breathing gas mixture change.

The decompression protocols were designed using V-planner (HHS Software Corp, Kingston, ON, Canada, v3.86). The bottom time was set to 20 min, and the decompression gas chosen was nitrox 50. The total dive duration calculated using V-planner was 58 min (the detailed diving protocol is provided in Figure 2).

The formation of venous gas bubbles was measured 30 min after the dive while resting and after performing two coughs. FMD was monitored before the dive and 40–60 min after the dive. All research procedures were approved by the Research Ethics Committee of the University of Split School of Medicine (reference number: 2181-198-03-04-14-0023) in agreement with the Declaration of Helsinki. The subjects were informed of all possible risks associated with their participation in the study before giving written consent.

### 2.2. Venous Bubble Monitoring during Post-Dive Observation

Ultrasonic measurement was conducted by two experienced cardiologists (DG and AO) 30 min post-dive using a phase array probe (1.5–3.3 MHz) of a Vivid Q ultrasonic scanner (GE Healthcare, Milwaukee, WI, USA). Divers were placed in a supine position, and gas bubbles were observed while resting and after two coughs as high-intensity echoes in the cardiac chambers. A modified scale created by Eftedal and Brubakk was used to grade detected bubble formation as follows: 0 = no bubbles, 1 = occasional bubbles, 2 = at least one bubble/fourth heart cycle, 3 = at least one bubble/heart cycle, 4 = continuous bubbling with at least one bubble/cm^2^ in all frames, and 5 = “white out” where individual bubbles cannot be observed [27].

### 2.3. Pre- and Post-Dive Endothelial Function Monitoring

Brachial artery flow-mediated vasodilation following reactive hyperemia was used to assess endothelial function as described previously [28]. The measurements were performed in a silent room at a temperature of 20 °C. The divers were placed in a supine position on a bed, and they rested for at least 10 min before any measurement was performed. A Vivid Q Expert ultrasonic scanner (5.7–13.3 MHz linear transducer) was used to determine flow-mediated vasodilatation before the dive and in the period between 40 and 60 min after the divers reached the surface. Longitudinal images were employed to measure the brachial artery diameter with simultaneous electrocardiogram recording. The onset of the R wave was used to determine end diastole. After careful selection, the borders of the images were positioned manually for diameter measurement with an electronic caliper. A pulsed Doppler system with a sample volume positioned in the central portion of the brachial artery was used to measure blood flow velocities. To accomplish this, the ultrasound probe was positioned over the same segment of the brachial artery, and its location was designated with a permanent marker. In most instances, measurement was performed 3–5 cm proximal to the antecubital fossa. The basal measurement was followed by a 5 min brachial artery occlusion. For that purpose, a manometer cuff was placed on the forearm of the subject and inflated to 240 mmHg to ensure the complete cessation of blood flow. After the cuff was deflated, a short but immense increase in blood flow was observed, which was followed by an increase in the arterial diameter caused by an increase in shear stress. The artery diameter and blood velocity were measured at 0, 30, 60, 90, 120, 150, 180, 240, and 300 s after the deflation of the cuff. FMD was calculated as the percentage increase in the brachial artery diameter from the resting state to the maximum dilatation. The ultrasound system’s internal memory was used to store images for later analysis [2]. We did not use nitroglycerine to measure endothelium-independent dilatation because it may interfere with gas bubble formation after diving [28,29]. The cardiologists in charge of the ultrasonic measurements were blinded to the diving protocol.

### 2.4. Cognitive and Psychomotor Performance Testing

The CRD series was used to measure cognitive and psychomotor performance. It consists of software and electronic instruments and provides 38 standardized tests. The CRD series does not depend on language proficiency or any other knowledge, and it can be used with various age groups. It can be used to repetitively test the same subject. Three representative tests were performed, from the simplest to the most complex, including (1) the test of convergent thinking (CRD11), measuring the speed of solving simple arithmetic operations; (2) the light signal position discrimination test (CRD311), measuring the speed of perception; and (3) the test of operative thinking (CRD411), measuring complex psychomotor coordination. Depending on the test, the psychometric assessment consisted of 60 (CRD311) or 35 (CRD11 and CRD411) individual tasks. When the divers gave the correct response to a single task, the following task was presented. In this manner, the tests were not time-restricted, and the completion time depended on the respondents. Hence, the average time for the completion of all 3 tests in this group of respondents was from 7 to 10 min, including the time it took the investigators to provide complete instructions to the respondents between the tests.

Testing was conducted in a silent, bright room before and 60 min after each dive under the supervision of an experienced researcher who was blinded to the divers’ protocol assignments. In order to avoid learning effects, the divers were tested multiple times until stable baseline results were reached. Six parameters were analyzed: total test solving time (TTST), minimum single-task solving time (MinT), median single-task solving time (MedT), start ballast (SB), end ballast (EB), and total ballast (TB). TTST, MinT, and MedT were descriptors of speed, accuracy, and mental endurance. SB, EB, and TB were indicators of the stability of psychomotor functioning during the process of testing. They were calculated as the sum of the differences between the time spent on each individual item (Ti) and the minimum single-task solving time in the first (SB) and second halves (EB) of the test and the total test (TB) [30].

### 2.5. Measurement of State and Trait Anxiety

An assessment of the anxiety of the divers was performed using the Croatian version of Spielberger, Gorsuch, and Lushene’s (1970) State-Trait Anxiety Inventory (STAI) [31]. This scale is suitable for people over 16 years old. The STAI was previously used to test anxiety in scuba divers and proved to be reliable [1,32,33]. Both scales consist of 20 items rated on a 4-point Likert scale ranging from 1 (not at all) to 4 (absolute agreement). The state scale can be further divided into 10 statements that are expressed in an anxiety-present direction (e.g., “I am worried”) and 10 items that are expressed in an anxiety-absent direction (e.g., “I feel calm”). The trait scale comprises seven items that are anxiety-absent and 13 anxiety-present statements. By adding all the scores, each scale has a total score between 20 and 80 [31].

On the day before each dive, the divers completed the STAI questionnaire and CRD testing (one series of practice exercises followed by a recorded test). After the dive, the participants’ FMD was measured, and bubbles were detected. Subsequently, the divers were taken to another room where they completed the STAI questionnaire and CRD tests (60–80 min after surfacing). The researcher that performed the cognitive and psychomotor performance testing and the measurement of the STAI was blinded to the diving protocol.

### 2.6. Statistical Analysis

**Figure 3:**
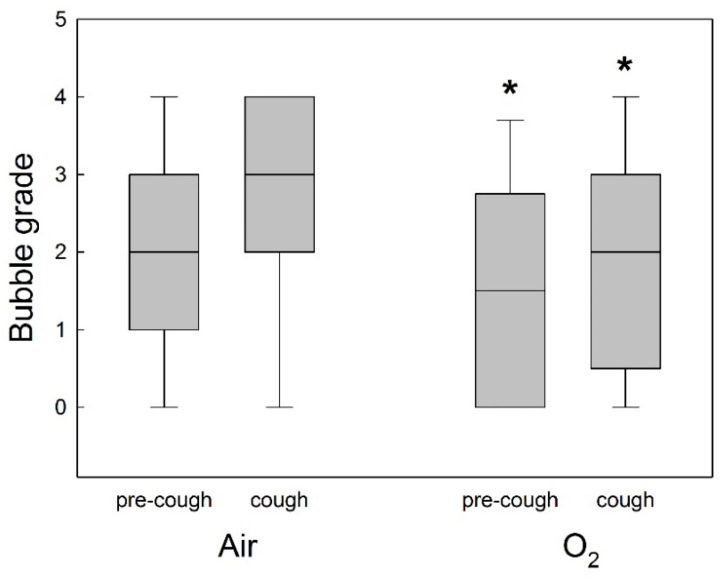
Post-dive circulating bubble detection for all divers after air or oxygen (O_2_) prebreathing in all experimental conditions (before or after a cough). Data are presented as medians (horizontal lines), 25th and 75th quartiles (shaded squared areas), and 10th and 90th percentile ranges (error bars) of the venous bubble grades measured after the dives. * Significant difference in bubble grade in O_2_ prebreathing protocol compared to air prebreathing protocol (*p* < 0.05).

The data were analyzed using MedCalc Statistical Software version 17.1 (MedCalc Software, Ostend, Belgium) and IBM SPSS Statistics for Windows Student Version 14.0 (IBM Corp., Armonk, NY, USA). Data are presented as means ± standard deviations (standard errors in Figure 3) or as medians and interquartile ranges. Different measures of central tendency and variability are reported depending on the results of the Shapiro–Wilk test for the normality of variables. When the results indicate an asymmetric distribution following the Shapiro–Wilk normality test as well as a visual inspection of the distribution of the results in Q-Q plots of the data, medians and interquartile ranges are reported instead of means ± standard deviations. The comparison of the bubble grade at rest and after a cough in the oxygen prebreathing group versus the air prebreathing group was performed using the non-parametric Wilcoxon signed-rank test. The differences between the pre-dive and post-dive FMD in both groups were reported using a parametric t-test for paired samples and were expressed as means and standard deviations. The significance of the Wilcoxon signed-rank tests was calculated for the comparison of brachial artery blood flow (mL/min) as well as to assess differences in psychomotor performance on the CRD-series tests and anxiety data from the STAI questionnaire. Bubble grades and psychomotor reaction times are presented as medians (25–75% quartile ranges), and they were compared using a non-parametric Friedman analysis of variance. Wilcoxon signed-rank tests determined the significance of all pairwise multiple comparisons, with the Bonferroni correction. The threshold of significance was set at *p* < 0.05.

## 3. Results

### 3.1. Participants

All divers performed two dives according to the protocol, and no divers showed any symptom of DCS. The divers reported that the dives lasted as long as planned (58 min) and that they were able to reach the designated depth (60 msw, Figure 2). Table 1 shows the demographic characteristics of the divers.

### 3.2. Post-Dive Monitoring and Venous Bubble Detection

In the O_2_ prebreathing group, the bubble grade was lower at rest and after a cough in comparison to the air prebreathing group (*p* < 0.001, Figure 3).

The bubble grade at rest (before a cough) for the air prebreathing protocol was 2 (1–3), and for the O_2_ prebreathing protocol this value was 1.5 (0–2.25), as measured 30 min after the dive (*p* < 0.005, Figure 3). The bubble grade after a cough for the air prebreathing protocol was 3 (2–4), and for the O_2_ prebreathing protocol this value was 2 (1.5–3), as measured 30 min after the cough (*p* = 0.035; Figure 3).

### 3.3. Flow-Mediated Dilatation and Sheer Rate

**Figure 4:**
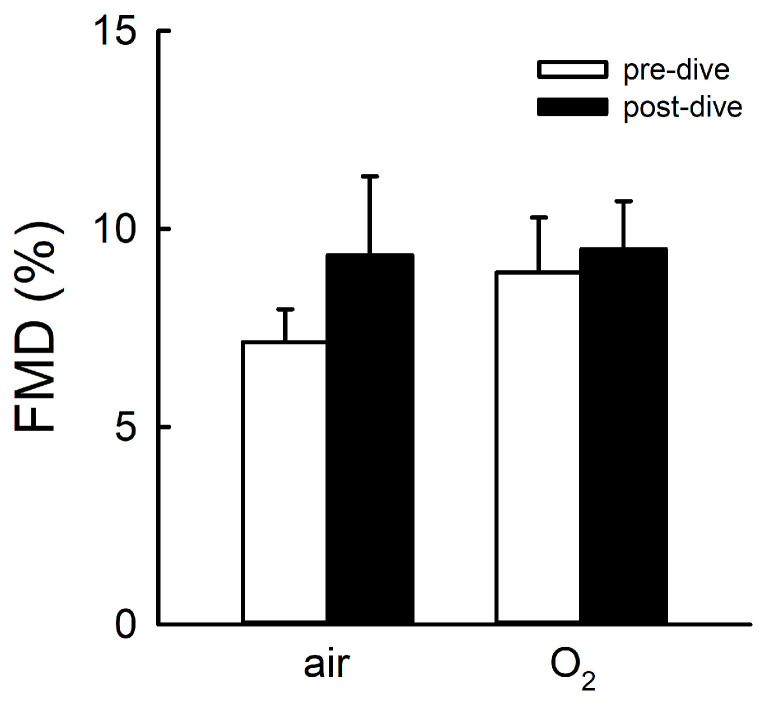
Flow-mediated vasodilatation (FMD) after air or O_2_ prebreathing protocols. FMD values are shown before and after trimix dives. The data are presented as means ± SDs (*p* < 0.05).

There was no significant difference between pre- and post-dive FMD in either group (*p* = 0.274). In the air prebreathing group, FMD was 7 ± 1% pre-dive vs. 9 ± 2% post-dive, whereas in the O_2_ prebreathing group FMD was 9 ± 1% pre-dive vs. 9 ± 1% post-dive (Figure 4).

Absolute values of brachial artery blood flow and brachial artery sheer rate prior to cuff inflation and during the 240 s after the release of the underarm cuff are shown in Table 2 and Table 3.

### 3.4. Psychomotor Performance

In the CRD311 test, there was no significant change in the speed of the simple reaction time, regardless of the composition of the gas mixture before the dive (TTST after air prebreathing trimix dive *p* = 0.273 vs. TTST after oxygen prebreathing trimix dive *p* = 0.249; Table 4).

In the CRD411 test, no difference was found in performance before and after the dive in divers when breathing air before the trimix dive (TTST *p* = 0.715, MinT *p* = 0.465, and MedT *p* = 0.715), while a faster complex psychomotor speed was found in divers when breathing oxygen 30 min before the trimix dive (TTST *p* = 0.028, MinT *p* = 0.046, and MedT *p* = 0.046; Table 4).

In the CRD11 test, the total reaction time was faster after the dive, independent of the gas mixture (air or oxygen) breathed 30 min before each dive (TTST trimix *p* = 0.028 and MedT *p* = 0.028; Table 4).

There was no statistically significant difference in anxiety as a state or trait, assessed using the STAI questionnaire, before and after trimix dives using air (*p* = 0.753 and *p* = 0.112, respectively) or oxygen 30 min before the dives (*p* = 0.207 and *p* = 0.339, respectively) (Table 5).

## 4. Discussion

The main finding of this study was that venous bubble formation was significantly reduced in the oxygen prebreathing protocol in comparison to the air prebreathing protocol after trimix dives to 60 msw. Additionally, endothelial function, measured via FMD, showed no significant difference between the protocols. The cognitive and psychomotor capabilities measured using the CRD-series tests prior to and after the dives showed faster complex psychomotor speeds (TTST, MinT, and MedT) in the CRD411 test when the divers were exposed to the oxygen prebreathing protocol. On the other hand, in the CRD11 test, the TTST and MedT were faster after the dives in both the air and oxygen prebreathing protocols. There was no difference in anxiety as a state or trait before and after the dives in both prebreathing protocols.

*Venous bubble reduction.* In this study, a noteworthy reduction in venous bubble formation was observed when the subjects engaged in O_2_ prebreathing prior to a trimix dive. This implies a favorable impact of oxygen in mitigating bubble formation after deep decompression dives. Previous research has established that factors such as individual susceptibility, dive-to-dive variations, and pre-dive preconditioning can contribute to the variability in bubble formation [34]. The role of oxygen-enriched air on bubble formation has been investigated in scuba diving, indicating that in both no-decompression diving protocols at 18 msw and decompression diving protocols at 45 msw, venous gas bubbling was decreased in nitrox (oxygen-enriched air) dives compared with air dives [9,35]. Additionally, the decrease in bubble formation was significant in dive protocols with 99% O_2_ [9]. Furthermore, the protective effect of increased O_2_ levels was shown in a study using O_2_ prior to dives [13]. It appears that breathing oxygen prior to diving may reduce bubble formation during dives, indicating that an overall reduction in the inert gas content or the removal of gas micronuclei before they create bubbles may be mechanisms supporting this phenomenon [13,36]. Additionally, a comparison of hyperbaric vs. normobaric oxygen prebreathing showed that hyperbaric pre-oxygenation was more effective in the reduction in bubble scores compared with normobaric pre-oxygenation, probably because it delivers more oxygen to the whole body for nitrogen elimination [37]. In our study, trimix was employed to facilitate research on deep technical dives at 60 msw, with a comparison of two prebreathing protocols. The results of this study are in accordance with earlier research related to oxygen prebreathing protocols, indicating a beneficial role of oxygen concerning bubble formation after deep decompression dives.

*No change in FMD.* In this study, endothelial function, measured via FMD, showed no significant difference regardless of the protocol groups. In our study, divers were exposed to a high oxygen pressure during the dives (1.4 bar at 60 msw), but no changes in FMD were observed when comparing the pre- and post-dive FMD. Increased oxygen pressure and its consequences on FMD have often been discussed from opposing standpoints [2,9]. The negative effects of diving related to endothelial dysfunction have been reported, but the exact mechanisms are still unknown [2,5,35]. In this study, bubbles were reduced in the oxygen prebreathing protocol, with no significant effect on FMD. The negative consequences of a higher bubble grade on arterial endothelial function are often seen as a larger FMD reduction, although an FMD reduction can occur even without direct contact with bubbles [2,5,35]. Additionally, the consequences of hyperoxia have been a topic of debate for many years, showing both the beneficial and detrimental effects of increased oxygen partial pressure [2,9]. Hyperoxia is believed to increase the production of reactive oxygen species (ROS), which can damage the endothelium and thus additionally contribute to microvascular dysfunction [15]. An additional mechanism that may lead to endothelial dysfunction is the formation of peroxynitrite anions (ONOO^−^). Peroxynitrite is formed rapidly when NO reacts with superoxide and is then slowly transformed into nitrogen dioxide (NO_2_) in reactions with molecular O_2_ [38]. These compounds can damage normal endothelial function. During and after diving, endothelial function can be influenced by different factors such as the depth of the dive, the number of successive dives, and the gas mixtures used for descent and ascent. It has been shown that acute endothelial dysfunction can occur in large conduit arteries after successive deep trimix dives, indicating possible cumulative effects [39]. Although oxygen prebreathing might have detrimental effects on FMD due to prolonged exposure to hyperoxia, we found no significant difference in FMD regardless of gas mixture preconditioning, indicating beneficial effects of oxygen prebreathing during trimix dives.

*Effect of O_2_ prebreathing on psychomotor performance.* It has been shown that elevated oxygenation and endothelial dysfunction could be associated with psychomotor and cognitive performance. Reductions in the speed of information processing, psychomotor skills, mental flexibility, and attention have been observed in individuals with type 2 diabetes and in older populations [25,26]. Cognitive performance in relation to the bubble formation detected via two-dimensional ultrasound is yet to be established, but endothelial dysfunction might lead to changes in psychomotor performance indirectly. Different gas mixtures with decreased N_2_ and increased O_2_ have been suggested to reduce the risk of N_2_ narcosis and the formation of N_2_ bubbles [13,40]. The protective effects of such mixtures on cognitive and psychomotor performance are still unclear. Previously, the protective effects of nitrox mixtures on both memory and alertness during scuba diving in comparison to air-breathing protocols were reported [40]. Reaction times in the underwater environment were evaluated in a study investigating working memory and executive functions while taking into account the cognitive effects induced by moderate physical exercise in underwater conditions [41]. In our study, psychomotor and cognitive performance were measured after the dives, showing no impairment in reaction time in all tests used after the trimix dives. Additionally, this study suggests that oxygen prebreathing may have had a protective effect on complex psychomotor speed.

*Influence of trimix diving on anxiety.* The anxiety experienced by scuba divers should also be taken into consideration when they perform different diving procedures. It is characterized by negative emotions that can influence divers’ physiological and cognitive performance, including worse cognitive performance in open water in comparison to a chamber, probably due to the uncertainty of being in an open-sea situation [23,24]. Furthermore, there is evidence that scuba diving performance and anxiety scores have a linear correlation; the best performance is related to a low level of anxiety, and the worst performance is related to a high anxiety level [32,42]. One should be mindful that although anxiety and panic attacks are more likely to affect less experienced divers, they can still occur in every diver, increasing the risk of DCI and other fatalities in scuba diving, which have been shown to be more probable in individuals with elevated levels of trait anxiety [31,33,43,44]. It might be speculated that the time of assessment could have influenced the results. The current study only aimed to assess the short-term effects of underwater dives using trimix gas mixtures with or without oxygen prebreathing. Long-term detrimental cognitive effects have previously been suggested in professional scuba divers [45]. The measurements of the technical divers in this study revealed no discernible adverse effects on psychomotor reaction time.

*Study limitations.* The sample is small, but this is not unusual for research involving similar protocols due to the limited number of divers certified for demanding diving [2,3,5,9]. The size of the sample was similar to that of an equivalent study [39]. Furthermore, due to the sex distribution of our cohort, the results are more applicable to the male population. The echocardiographic detection of bubbles following the dives in our study was performed both after rest and after a cough as a provocative maneuver. Resting and provocation bubble measurements can produce distinctly different results because active provocations can promote showers of detectable bubbles. Since our results may have been impacted by the provocative maneuver chosen for the peak bubble grade, we expressed and reported both measurements in this manuscript. The divers used their diving computers set to a bottom timer and reported whether the dives were executed in accordance with the plan determined using V-planner rather than reporting the actual depth achieved and the duration of the dive. The divers were not blinded in regard to which gas they breathed prior to the dives, which might have influenced the anxiety levels of the divers. However, the results show that there was no difference in the anxiety level before and after the dive when using either air or oxygen 30 min before the dive.

*Insights for the future.* Future studies might investigate different preconditioning procedures such as the duration of O_2_ prebreathing; its relation to the beginning of the dive; or breathing a mixture with a smaller percentage of O_2_, which may exert fewer toxic effects.

## 5. Conclusions

Bubble formation was reduced in the oxygen prebreathing protocol for trimix dives, with no significant change in FMD in either protocol, suggesting decreased bubble formation and the preservation of endothelial function in the oxygen prebreathing protocol in our study. Additionally, there was no psychomotor impairment after the dives in both prebreathing protocols. A faster complex psychomotor speed was seen after the dives in divers performing the oxygen prebreathing protocol, suggesting protective effects of oxygen exposure on the cognitive and psychomotor performance of divers. This could be taken into consideration when selecting further diving protocols.

## Figures and Tables

**Table 1:** Demographic characteristics of participants.

Parameter (Mean ± SD)	
Number	12
Gender (male/female)	11/1
Age (years)	37 ± 8
Body mass (kg)	84.6 ± 16.2
Height (cm)	179 ± 7
Body mass index (kg/m^2^)	26.2 ± 3.4
Blood pressure (mmHg)	
Systolic	131 ± 14
Diastolic	84 ± 8

**Table 2:** Comparison of pre-dive and post-dive measurements of brachial artery blood flow in air and O_2_ prebreathing protocols.

		Brachial Artery Blood Flow (mL/min)					
		Air Prebreathing			O_2_ Prebreathing		
		Pre-Dive *	Post-Dive ^†^	*p*	Pre-Dive ^‡^	Post-Dive ^‖^	*p*
Time after deflation	Pre-cuff	356 (312–414)	436 (345–516)	0.424	373 (297–463)	411 (304–499)	0.657
	0″	1013 (888–1197)	1072 (825–1333)	0.530	1050 (835–1334)	1244 (784–1462)	0.814
	30″	714 (571–902)	822 (720–894)	0.050	918 (590–1068)	851 (737–1053)	0.937
	60″	560 (467–780)	680 (523–830)	0.158	656 (526–832)	706 (602–873)	0.239
	90″	495 (420–585)	569 (507–691)	0.015	610 (437–738)	671 (488–736)	0.272
	120″	402 (382–598)	607 (426–686)	0.060	521 (424–585)	606 (504–706)	0.136
	150″	444 (378–602)	544 (470–677)	0.347	557 (434–638)	641 (503–697)	0.155
	180″	437 (363–603)	512 (427–701)	0.480	554 (366–653)	589 (511–662)	0.347
	240″	438 (335–572)	453 (406–590)	0.480	469 (355–687)	554 (464–613)	0.937

Wilcoxon signed-rank test significance reported for each pre–post comparison. * Friedman repeated-measures analysis of variance: χ^2^ = 64.93; *p* < 0.001. Values at 0″, 30″, and 60″ were significantly different from pre-cuff value following Bonferroni correction (*p* < 0.05). ^†^ Friedman repeated-measures analysis of variance: χ^2^ = 76.33; *p* < 0.001. Values at 0″, 30″, 60″, and 90″ were significantly different from pre-cuff value following Bonferroni correction (*p* < 0.05). ^‡^ Friedman repeated-measures analysis of variance: χ^2^ = 64.47; *p* < 0.001. Values at 0″, 30″, 60″, and 90″ were significantly different from pre-cuff value following Bonferroni correction (*p* < 0.05). ^‖^ Friedman repeated-measures analysis of variance: χ^2^ = 72.73; *p* < 0.001. Values at 0″, 30″, 60″, and 90″ were significantly different from pre-cuff value following Bonferroni correction (*p* < 0.05).

**Table 3:** Comparison of pre-dive and post-dive measurements of brachial artery sheer rates in air and O_2_ prebreathing protocols.

		Brachial Artery Shear Rate (mL/min)					
		Air Prebreathing			O_2_ Prebreathing		
		Pre-Dive *	Post-Dive ^†^	*p*	Pre-Dive ^‡^	Post-Dive ^‖^	*p*
Time after deflation	Pre-cuff	90 (78–114)	107 (86–121)	0.286	91 (74–112)	86 (75–105)	0.722
	0″	227 (214–271)	227 (180–285)	0.695	223 (197–258)	229 (197–269)	0.480
	30″	150 (132–181)	170 (137–207)	0.213	174 (153–201)	165 (137–201)	0.937
	60″	111 (104–143)	142 (97–163)	0.239	128 (106–147)	132 (96–146)	0.209
	90″	102 (83–119)	121 (93–133)	0.071	106 (98–130)	114 (96–130)	0.308
	120″	96 (84–108)	118 (90–136)	0.099	92 (86–111)	108 (89–121)	0.272
	150″	96 (85–115)	105 (94–138)	0.388	101 (89–117)	109 (92–121)	0.424
	180″	98 (85–114)	104 (87–138)	0.638	97 (89–111)	105 (92–128)	0.530
	240″	93 (75–121)	94 (81–132)	0.875	96 (84–125)	101 (85–111)	0.754

Wilcoxon signed-rank test significance reported for each pre–post comparison. * Friedman repeated-measures analysis of variance: χ^2^ = 56.15; *p* < 0.001. Values at 0″ and 30″ were significantly different from pre-cuff value following Bonferroni correction (*p* < 0.05). ^†^ Friedman repeated-measures analysis of variance: χ^2^ = 64.47; *p* < 0.001. Values at 0″, 30″, and 60″ were significantly different from pre-cuff value following Bonferroni correction (*p* < 0.05). ^‡^ Friedman repeated-measures analysis of variance: χ^2^ = 56.67; *p* < 0.001. Values at 0″, 30″, and 60″ were significantly different from pre-cuff value following Bonferroni correction (*p* < 0.05). ^‖^ Friedman repeated-measures analysis of variance: χ^2^ = 68.47; *p* < 0.001. Values at 0″, 30″, and 60″ were significantly different from pre-cuff value following Bonferroni correction (*p* < 0.05).

**Table 4:** Changes in psychomotor performance on CRD-series tests before and after trimix dives with divers breathing air or oxygen 30 min before the dives.

		Air Prebreathing			O_2_ Prebreathing		
		Pre-Dive	Post-Dive	*p*	Pre-Dive	Post-Dive	*p*
Light signal position discrimination test (CRD311)	TTST	33 (30–35)	31 (29–33)	0.273	28 (25–30)	25 (24–29)	0.249
	MinT	0.3 (0.3–0.4)	0.4 (0.3–0.4)	0.465	0.3 (0.2–0.3)	0.3 (0.2–0.3)	0.249
	MedT	0.5 (0.5–0.6)	0.5 (0.4–0.5)	0.273	0.5 (0.4–0.5)	0.4 (0.4–0.5)	0.345
Complex psychomotor coordination test (CRD411)	TTST	28 (28–30)	31 (26–35)	0.715	31 (24–34)	25 (21–28)	0.028 *
	MinT	0.4 (0.4–0.5)	0.4 (0.4–0.5)	0.465	0.4 (0.4–0.5)	0.3 (0.3–0.4)	0.046 *
	MedT	0.6 (0.6–0.7)	0.7 (0.6–0.7)	0.715	0.7 (0.6–0.7)	0.6 (0.5–0.6)	0.046 *
Simple arithmetic operations test (CRD11)	TTST	118 (107–127)	106 (90–122)	0.028 *	102 (92–115)	87 (82–108)	0.028 *
	MinT	2 (2–2)	2 (2–2)	0.753	2 (1–2)	2 (1–2)	0.075
	MedT	3 (3–3)	3 (3–3)	0.028 *	3 (2–3)	2 (2–3)	0.028 *

* Significant difference in reaction time when pre-dive and post-dive times were compared. Abbreviations: TTST—total test solving time, MinT—the best (shortest) single-task solving time, MedT—median time to solve task.

**Table 5:** Changes in anxiety as a state and trait before and after trimix dives with divers breathing air or oxygen 30 min before the dives.

	Air Prebreathing			O_2_ Prebreathing		
	Before the Dive	After the Dive	*p*	Before the Dive	After the Dive	*p*
Anxiety as a state (STAI-S)	26 (23–27)	25 (21–30)	0.753	30 (25–33)	25 (22–31)	0.207
Anxiety as a trait (STAI-T)	31 (27–33)	27 (25–29)	0.112	29 (27–38)	31 (26–36)	0.339

## Data Availability

The data that support the findings of this study are available from the corresponding author upon reasonable request. The data are not publicly available due to participant privacy.
